# Murine leukemia virus targets innate-like B1 B cells to establish infection in mice

**DOI:** 10.1186/1742-4690-10-S1-P84

**Published:** 2013-09-19

**Authors:** Xaver Sewald, Christin Herrmann, Fei Li, Walther Mothes

**Affiliations:** 1 Yale University, Department of Microbial Pathogenesis, New Haven, CT 06536, USA

## Background

Retroviruses are believed to efficiently spread via sites of cell-cell contact designated virological synapses. This model was developed based on *in vitro* evidence in which infected cells establish cell-cell contact with uninfected cells. Applying intravital microscopy, we were recently able to provide *in vivo* support for the existence of virological synapses within the lymph node of living mice. Visualizing cells infected with fluorescently labeled murine leukemia virus (MLV) we identified B cells that were able to form long-lived virological synapses with uninfected lymphocytes [1]. *In vivo* virological synapses were, like their *in vitro* counterpart, dependent on the expression of the viral envelope glycoprotein (Env) and characterized by a prolonged polarization of viral capsid to the cell-cell interface. B cells were among the first cells to become infected by incoming MLV. However, the specific subtype of B cells that is susceptible to MLV had remained unknown.

## Results

Here we present evidence for a critical role of innate-like B1 B cells in the establishment of MLV infection in mice. Adoptively transferred B1 B cells are selectively targeted by MLV. Mice lacking B1 B cells are resistant to MLV infection. In addition, using knockout mice we provide evidence for the contribution of adhesion factors expressed by B1 B cells in spreading of retroviruses *in vivo.*

## Conclusions

Our work reveals the critical importance of a distinct B cell subset in the susceptibility to retroviral infections under physiological conditions *in vivo.*
